# Design and synthesis of new esters of terpenoid alcohols as 15-lipoxygenase inhibitors

**DOI:** 10.22038/IJBMS.2018.27910.6794

**Published:** 2018-07

**Authors:** Hamid Sadeghian, Seyed Mohammad Seyedi, Zeinab Jafari

**Affiliations:** 1 Neurogenic Inflammation Research Center, Mashhad University of Medical Sciences, Mashhad, Iran; 2 Department of Laboratory Sciences, School of Paramedical Sciences, Mashhad University of Medical Sciences, Mashhad, Iran; 3 Department of Chemistry, Faculty of Sciences, Ferdowsi University of Mashhad, Mashhad, Iran

**Keywords:** Inhibitors, Phenolic acid, Radical scavenging, Terpenoids, 15-lipoxygenase

## Abstract

**Objective(s):**

15-Lipoxygenases are one of the iron-containing proteins capable of performing peroxidation of unsaturated fatty acids in animals and plants. The critical role of enzymes in the formation of inﬂammations, sensitivities, and some cancers has been demonstrated in mammals. The importance of enzymes has led to the development of mechanistic studies, product analysis, and synthesis of inhibitors.

**Materials and Methods:**

The inhibitory activity of all synthetic compounds against SLO (soybean 15-lipoxygenase: L1; EC 1,13,11,12) was determined using the peroxide formation method. In this method, the basis of evaluation of lipoxygenase activity is measuring the concentration of fatty acid peroxide. All measurements were compared with 4-​methyl-​2-​(4-​methylpiperazinyl)pyrimido[4,​5-​b]benzothiazine (4-MMPB) as one of the known lipoxygenase inhibitors. The radical scavenging ability of all synthetic compounds using stable free radicals (DPPH: 2,2-diphenyl-1-picrylhydrazyl) was measured for further investigation.

**Results:**

In this study, a series of esters from phenolic acids with terpenoid alcohols was synthesized and their inhibitory potency against soybean 15-lipoxygenase and their free radical scavenging properties were determined. Among the synthetic compounds, adamantyl protocatetuate 2j and bornyl protocatetuate 2o showed the most potent inhibitory activity with IC50 values of 0.95 and 0.78 μm, respectively.

**Conclusion:**

By changing the alcohol and acyl portions of stylosin, it was found that electronic properties play main role in lipoxygenase inhibition potency in contrast with steric features. Insertion of more reductive phenolic moiety such as catechuate and gallate lead to more lipoxygenase inhibition potency of the esters as observed in their radical scavenging activity.

## Introduction

**Figure 1:**
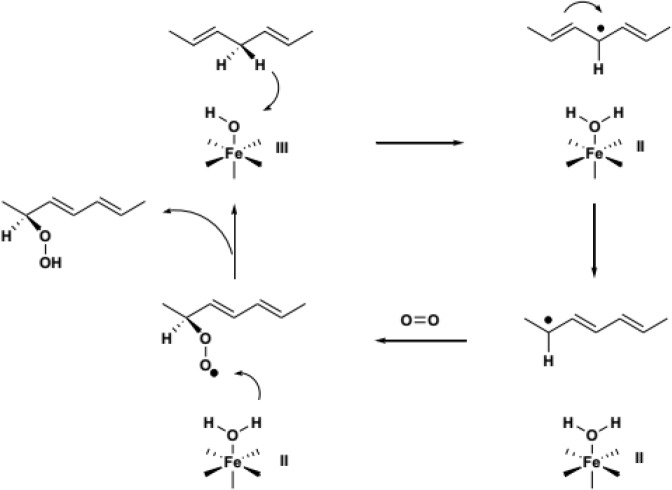
Enzymatic cycle of peroxidation of unsaturated fatty acids

Mammalian lipoxygenases are iron-bound enzymes that are responsible for the oxidation of unsaturated fatty acids and esters to hydroproxy derivatives (Figure 1). Enzymes of this group have been widely found in plants and animals and are also named based on the situation where a key substrate, such as arachidonic acid and or other unsaturated fatty acids containing a cis, cis-1,4-pentadiene moiety, is oxidized (1). Based on the results of recent research, 15-lipoxygenases have been introduced as an interesting target for interfering treatment of some diseases (2). It has been shown that 15-lipoxygenases are effective in the progression of certain cancers (3-6). In recent years, findings have been discovered based on the inhibitory effect of the 15-lipoxygenase enzymes in cardiovascular disease treatment (7). Researches have shown that products derived from arachidonic acid and linoleic acid oxidation by 15-lipoxygenases can be used as pre-inflammation and pre-anorexia agents (8). Three main pathways have been identified that inhibit the activity of lipoxygenase: (1) Reducing inhibitors or antioxidants that interact with the reduction of 15-lipoxygenase. 2) Complexing iron-fixing agents, 3) Non-reducing competitive inhibitors, which compete with arachidonic acid or other unsaturated fatty acids containing a cis, cis-1,4-pentadiene moiety in order to bind to the active site of the enzyme (9). 

Natural compounds are a source of lipoxygenase enzyme inhibitors (10). Phenolic compounds such as catechols and cinnamic acid derivatives, which have hydroxyl groups, are appropriate inhibitors of lipoxygenases due to their antioxidant properties. Caffeic acid has been shown to act as an inhibitor of the lipoxygenase enzyme (11). Stylosin, which was extracted from ferula plant for the first time, is a natural monoterpenoid compound that consists of two parts: monoteropen (fenchol) and vanillic acid (12). Studies conducted on its inhibitory power against the 15-lipoxygenase enzymes showed that this compound has an appropriate activity. According to this finding, we decided to synthesize a series of stylosin- based ester compounds and investigate their inhibitory activity as well as their free radical scavenging properties.

## Materials and Methods

***15- lipoxygenase inhibitory assessment***

Linoleic acid and two assay solutions (A and B) were prepared in advance. Solution A was 50 mM (3-dimethylaminobenzoic acid) (DMAB) in l00 mM phosphate buffer (pH 7.0). Solution B was a mixture of l0 mM (3-methyl-2-benzothiazolonhydrazone) (MBTH) (3 ml), hemoglobin (5 mg/ml, 3 ml) in 50 mM phosphate buffer at pH 5.0 (25 ml). A linoleic acid solution was prepared by mixing 5 mg of linoleic acid with 0.5 ml ethanol and then diluting with KOH 100 mM to a final volume of 5 ml. In the standard assay, the sample in ethanol (25 µl), SLO (4,000 units/ml in 50 mM phosphate buffer pH 7.0; 25 µl), and phosphate buffer pH 7.0 (50 mM; 900 µl) were mixed in a test tube, and pre-incubation was carried out for 5 min at room temperature. A control test was performed with the same volume of ethanol. After pre-incubation, linoleic acid solution (50 µl) was added to start the peroxidation reaction, and, 7 min later, solution A (270 µl) and then solution B (130 µl) were added to start the color formation. Further, 5 min later, 200 µl of a 2 % sodium dodecyl sulfate (SDS) solution was added to terminate the reaction. The absorbance at 598 nm was compared to control test.

***Determination of DPPH bleaching***

DPPH solution (25 μM) in absolute ethanol was prepared. This solution was added to an equal volume of the solution of the test compounds (dissolved in ethanol) to obtain a desired concentration (50, 10, 2 and 0.4 µM). Ethanol was used as control solution. After 30 min at room temperature, the absorbance was recorded at 517 nm. 

***Experimental***

*Instrument*

Melting points of the products were determined with an Electrothermal Type 9100 melting point apparatus. The FT-IR spectra were recorded on pressed KBr pellets using an AVATAR 370 FT-IR spectrometer (Therma Nicolet spectrometer, USA) at room temperature in the range between 4000 and 400 cm-1 with a resolution of 4 cm-1. The NMR spectra were provided by Brucker Avance 300, 400 and 500 MHz instruments in deuterochloroform (CDCl_3_) and deuterated dimethyl sulfoxide (DMSO-*d*_6_) in the presence of tetramethylsilane as the internal standard, and the coupling constants (*J* values) are given in Hz. Elemental analyses were performed using a Thermo Finnigan Flash EA 1112. Mass spectra were recorded with a CH7A Varianmat Bremem instrument at 70 eV electron impact ionization, in m**/**z (rel%).

*General Procedure for Preparation of ester compounds (2a-p)*

N,N’-Dicyclohexylcarbodiimide (DCC) (2 mmol) was added to the solution of carboxylic acid (1.5 mmol) and alcohol (1.5 mmol) in dry THF (10 ml) at 0 ^°^C. After about 5 min, the solution was stirred overnight at room temperature. The reaction mixture was filtered off, the solvent was removed *in vaccum *and extracted with ethyl acetate (EtOAc; 3×10 ml), washed with diluted aqueous citric acid solution (20 ml), saturated aqueous NaHCO_3_ solution (20 ml) and water (15 ml), dried over MgSO_4_ and the crude residue was purified by column chromatography on silica gel (18).

*(2S)-1,3,3-trimethylbicyclo[2.2.1]heptan-2-yl 4-hydroxy-3-methoxybenzoate (2a)*

White solid, 20% yield; mp: 120-122^°^C; ^1^H NMR (400 MHz, CDCl_3_): δ=0.85 (s, 3H, H-9), 1.12 (s, 3H, H-8), 1.19 (s, 3H, H-10), 1.20-1.24 (m, 1H, H-6), 1.25-1.28 (dd, *J*_1_*=*10.4 Hz, *J*_2_*=*1.2 Hz, 1H, H-7), 1.49-1.57 (m, 1H, H-5), 1.66-1.69 (m, 1H, H-7), 1.76-1.83 (m, 2H, H-4, H-5), 1.89-1.97 (m, 1H, H-6), 3.96 (s, 3H, -OCH_3_), 4.60 (d, *J=*2 Hz, 1H, H-2), 6.05 (brs, 1H, OH), 6.97 (d, *J=*8 Hz, 1H, H-5’), 7.60 (d, *J=*1.6 Hz, 1H, H-2’), 7.67-7.69 (dd, *J*_1_*=*8 Hz, *J*_2_*=*1.6 Hz, 1H, H-6’); ^13^C NMR (100 MHz, CDCl_3_): δ= 19.53, 20.32, 25.93, 26.91, 29.74, 39.84, 41.45, 48.41, 48.61, 56.04, 86.48, 111.74, 114.00, 122.83, 123.96, 146.19, 149.86, 166.75; IR (KBr): 3374, 2950, 2876, 1688 cm^−1^; MS (*m/z*): 304 (M^+^); Anal. Calcd for (C_18_H_24_O_4_): C (71.03), H (7.95); found: C (70.89), H (7.63);

*(2S)-1,3,3-trimethylbicyclo[2.2.1]heptan-2-yl 3,4-dihydroxybenzoate(2b)*

White solid, 22% yield; mp: 58-61^°^C; ^1^H NMR (400 MHz, CDCl_3_): δ=0.85 (s, 3H, H-9), 1.11 (s, 3H, H-8), 1.18 (s, 3H, H-10), 1.22-1.3 (m, 2H, H-6, H-7), 1.49-1.55 (m, 1H, H-5), 1.67 (d, *J=*10 Hz, 1H, H-7), 1.75-1.79 (m, 2H, H-4, H-5), 1.89-1.92 (m, 1H, H-6), 4.59 (s, 1H, H-2), 6.68 (brs, 2H, -OH), 6.95 (d, *J=*8 Hz, 1H, H-5’), 7.61 (d, *J=*8 Hz, 1H, H-6’), 7.79 (s, 1H, H-2’);^ 13^C NMR (100 MHz, CDCl_3_): δ= 19.40, 20.36, 25.87, 26.87, 29.78, 39.91, 41.44, 48.42, 48.66, 87.14, 114.83, 116.76, 122.64, 123.61, 143.33, 149.07, 167.72; IR (KBr): 3476, 3431, 3366, 2958, 2929, 2870, 1688 cm^−1^; MS (*m/z*): 290 (M^+^); Anal. Calcd for (C_17_H_22_O_4_): C (70.32), H (7.46); found: C (70.05), H (7.24);

*(2S)-1,3,3-trimethylbicyclo[2.2.1]heptan-2-yl 3-methoxybenzoate (2c)*

Light yellow liquid, 30% yield; ^1^H NMR (400 MHz, CDCl_3_): δ=0.87 (s, 3H, H-9), 1.13 (s, 3H, H-8), 1.2 (s, 3H, H-10), 1.22-1.25 (m, 1H, H-6), 1.25-1.3 (dd, *J*_1_*=*10.3 Hz, *J*_2_*=*1.6 Hz, 1H, H-7) 1.47-1.58 (m, 1H, H-5), 1.67-1.70 (dq,* J*_1_=7.8 Hz, *J*_2_=2 Hz, 1H, H-7), 1.77-1.83 (m, 2H, H-4, H-5), 1.91-1.99 (m, 1H, H-6), 3.87 (s, 3H, -OCH_3_), 4.635 (d, *J=*1.2 Hz, 1H, H-2), 7.10-7.13 (ddd, *J*_1_*=*8.39 Hz,* J*_2_*=*2.8 Hz, *J*_3_*=*0.8 Hz, 1H, H-4’), 7.37 (t, *J=*8 Hz, 1H, H-5’), 7.61-7.62 (q, *J=*1.2 Hz, 1H, H-2’), 7.67-7.70 (dt, *J*_1_*=*6.4 Hz, *J*=1.2 Hz, 1H, H-6’) ; ^13^C NMR (100 MHz, CDCl_3_): δ= 19.49, 20.30, 25.92, 26.89, 29.75, 39.85, 41.46, 48.35, 48.63, 55.38, 86.80, 114.28, 119.00, 121.87, 129.39, 132.04, 159.57, 166.75; IR (KBr): 2839, 2871, 2957, 1719 cm^−1^; MS (*m/z*): 288 (M^+^); Anal. Calcd for (C_18_H_24_O_3_): C (74.97), H (8.39); found (%): C (73.60), H (8.10);

*(2S)-1,3,3-trimethylbicyclo[2.2.1]heptan-2-yl 3,4-dimethoxybenzoate (2d)*

Light yellow liquid, 25% yield; ^1^H NMR (300MHz, CDCl_3_): δ=0.84 (s, 3H, H-9), 1.12 (s, 3H, H-8), 1.2 (s, 3H, H-10), 1.21-1.28 (m, 2H, H-6, H-7), 1.45-1.57 (m, 1H, H-5), 1.60-1.69 (m, 1H, H-7), 1.71-1.82 (m, 2H, H-4, H-5), 1.86-1.96 (m, 1H, H-6), 3.89 (s, 3H, -OCH_3_), 4.601 (d, *J=*2.99 Hz, 1H, H-2), 6.92 (d, *J=*8.4 Hz, 1H, H-5’), 7.25 (d, *J=*1.2 Hz, 1H, H-2’), 7.72-7.75 (dd, *J*_1_*=*8.4 Hz, *J*_2_*=*1.2 Hz, 1H, H-6’); ^13^C NMR (75 MHz, CDCl_3_): δ= 18.90, 20.19, 25.84, 26.52, 29.82, 39.50, 41.16, 48.16, 48.63, 55.80, 55.96, 86.49, 110.24, 112.00, 123.25, 148.65, 152.86, 166.54; IR (KBr): 2955, 2871, 1710 cm^−1^; MS (*m/z*): 318 (M^+^); Anal. Calcd for (C_19_H_26_O_4_): C (71.67), H (8.23); found: C (70.39), H (8.05);

*(2S)-1,3,3-trimethylbicyclo[2.2.1]heptan-2-yl 3,4,5-trihydroxybenzoate (2e)*

Brown solid, 15% yield; mp: 171-173^°^C; ^1^H NMR (300 MHz, DMSO-*d*_6_): δ=0.75 (s, 3H, H-9), 1.05 (s, 3H, H-8), 1.11 (s, 3H, H-10), 1.14-1.19 (m, 1H, H-6), 1.22 (d,* J=*9 Hz, 1H, H-7), 1.42-1.55 (m, 1H, H-5), 1.65 (d, *J=*9 Hz, 1H, H-7), 1.69-1.77 (m, 2H, H-4, H-5), 1.83-1.92 (m, 1H, H-6), 4.42 (s, 1H, H-2), 7.00 (s, 2H, H-2’, H-6’), 8.97-9.33 (brs, 3H, -OH);^ 13^C NMR (75 MHz, DMSO-*d*_6_): δ= 19.76, 20.57, 25.97, 26.91, 29.95, 39.84, 41.24, 48.25, 48.53, 85.14, 108.9, 120.16, 138.64, 145.88, 166.59; IR (KBr): 3420, 2951, 2870, 1681 cm^−1^; MS (*m/z*): 307 (M^+^); Anal. Calcd for (C_17_H_22_O_5_): C (66.65), H (7.24); found (%): C (66.4), H (7.07);

*(2S)-1,3,3-trimethylbicyclo[2.2.1]heptan-2-yl(E)-3-(4-hydroxy-3-methoxyphenyl)acrylate (2f)*

Yellow liquid, 17% yield; ^1^H NMR (300 MHz, CDCl_3_): δ=0.85 (s, 3H, H-9), 1.11 (s, 3H, H-8), 1.18 (s, 3H, H-10), 1.20-1.26 (m, 1H, H-6), 1.23-1.26 (dd, *J*_1_*=*10.5, *J*_2_*=*1.5, 1H, H-7), 1.45-1.57 (m, 1H, H-5), 1.63-1.67 (dq,* J*_1_= 10.5 Hz,* J*_2_= 2 Hz,1H, H-7), 1.73-1.81 (m, 2H, H-4, H-5), 1.84-1.93 (m, 1H, H-6), 3.97 (s, 3H, -OCH_3_), 4.52 (d, *J=*1.8 Hz, 1H, H-2), 5.9 (brs, 1H, -OH), 6.36 (d, *J=*15.9 Hz, 1H, ArCH=C*H*CO), 6.95 (d, *J=*8.1 Hz, 1H, H-5’), 7.07 (d, *J* =1.8 Hz, H-2’), 7.10-7.13 (dd, *J*_1_*=*8.1 Hz, *J*_2_*=*1.8 Hz, 1H, H-6’), 7.63 (d, *J=*15.9 Hz, 1H, ArC*H*=CHCO); ^13^C NMR (75 MHz, CDCl_3_): δ= 19.45, 20.22, 25.91, 26.79, 29.77, 39.72, 41.49, 48.43, 55.59, 86.1, 109.3, 114.69, 115.94, 123.09, 127.14, 144.37, 146.77, 147.86, 167.72; IR (KBr): 3538, 3401, 2928, 2853, 1704 cm^−1^; MS (*m/z*): 330 (M^+^); Anal. Calcd for (C_20_H_26_O_4_): C (72.70), H (7.93); found: C (72.07), H (7.40);

*(2S)-1,3,3-trimethylbicyclo[2.2.1]heptan-2-yl (E)-3-(3,4-dihydroxyphenyl)acrylate (2g)*

Yellow liquid, 15% yield; ^1^H NMR (400 MHz, CDCl_3_): δ=0.84 (s, 3H, H-9), 1.11 (s, 3H, H-8), 1.17(s, 3H, H-10), 1.21-1.41(m, 2H, H-6, H-7), 1.41-1.58 (m, 1H, H-5), 1.58-1. 70 (m, 1H, H-7), 1.70-1.81 (m, 2H, H-4, H-5), 1.84-2.00 (m, 1H, H-6), 4.51 (s, 1H, H-2), 6.33 (d, *J=*16 Hz, 1H, ArCH=C*H*CO), 6.93 (d, *J=*8.0 Hz, 1H, H-5’), 7.04 (d, *J*= 8.4 Hz, 1H, H-6’), 7.17(s, 1H, H-2’), 7.6 (d, *J=*16, 1H, ArC*H*=CHCO);^ 13^C NMR (100 MHz, CDCl_3_): δ= 19.47, 20.22, 25.89, 26.76, 29.73, 39.76, 41.47, 48.42, 48.46, 55.59, 86.42, 114.34, 115.46, 122.38, 127.57, 143.95, 144.60, 146.45, 168.64; IR (KBr): 3534, 3349, 2953, 2925, 2872, 1678 cm^−1^; MS (*m/z*), 316 (M^+^); Anal. Calcd for (C_19_H_24_O_4_) calc. (%): C (72.13), H (7.65); found (%): C (71.80), H (7.40);

*(2R)-1,7,7-trimethylbicyclo[2.2.1]heptan-2-yl 4-hydroxy-3-methoxybenzoate(2h)*

White solid, 27% yield; mp: 82- 85^°^C (lit. 80-82 ^°^C) (19).

*(3s,5s,7s)-adamantan-1-yl 4-hydroxy-3-methoxy benzoate*

*(2i)*

White solid , 15% yield; mp: 136-139^°^C; ^1^H NMR (400 MHz, CDCl_3_): δ=1.72 (t, *J*=7.2 Hz, 6H(adamantly)), 2.24-2.22 (m, 9H (adamantyl)), 3.96 (s, 3H, -OCH_3_), 5.99 (brs, 1H, -OH), 6.94 (d, *J=*8.1 Hz, 1H, H-5’), 7.53 (d, *J*=1.2, 1H, H-2’), 7.59-7.61 (dd,* J*_1_*=*8.1 Hz, *J*_2_*=*1.2 Hz, 1H, H-6’); ^13^C NMR (100 MHz, CDCl_3_): 30.91, 36.12, 41.48, 56.01, 80.79, 111.51, 113.81, 123.51, 124.11, 146.04, 149.20, 165.36; IR (KBr): 3314, 2923, 2890, 1684 cm^−1^; MS (*m/z*): 302 (M^+^); Anal. Calcd for (C_18_H_22_O_4_) calc. (%): C (71.50), H (7.33); found (%): C (70.98), H (6.87);

*(3s,5s,7s)-adamantan-1-yl 3,4-dihydroxybenzoate(2j)*

White solid, 18% yield; mp: 170-173^°^C; ^1^H NMR (300 MHz, CDCl_3_): δ=1.4-2 (m, 6H, (adamantyl)), 2.00-2.26 (m, 9H, (adamantyl)), 5.81 (brs, 2H, -OH), 6.91 (d, *J=*8.1 Hz, 1H, H-5’), 7.5 (d, *J=*8.1 Hz, 1H, H-6’), 7.644 (s, 1H, H-2’); ^13^C NMR (75 MHz, CDCl_3_): δ= 30.72, 30.91, 35.97, 36.22, 41.48, 45.07, 81.47, 114.6, 116.58, 123.39, 124.08, 143.36, 148.79, 166.29; IR (KBr): 3326, 2922, 2989, 2870, 1684 cm^−1^; MS (*m/z*): 288 (M^+^); Anal. Calcd for (C_17_H_20_O_4_) calc. (%): C (70.81), H (6.99); found (%): C (70.14), H (6.40);

*(3s,5s,7s)-adamantan-1-yl 3,4,5-trihydroxybenzoate(2k)*

Brown solid, 10% yield; mp: 195-199^°^C; ^1^H NMR (300 MHz, DMSO-*d*_6_): δ=1.66 (s, 6H, (adamantly)), 2.15 (s, 9H, (adamantly)), 6.83 (s, 2H, H-2’, H-6’), 9.18 (brs, 3H, -OH); ^13^C NMR (75 MHz, DMSO-*d*_6_): δ= 30.67, 36.15, 41.43, 79.77, 108.83, 121.60, 138.73, 145.70, 165.72; IR (KBr): 3360, 3294, 2915, 2848,1678 cm^−1^; MS (*m/z*): 304 (M^+^); Anal. Calcd for (C_17_H_20_O_5_) calc. (%): C (67.09), H (6.62); found (%): C (66.6), H (6.28);

*(3s,5s,7s)-adamantan-1-yl (E)-3-(3,4-dihydroxyphenyl) acrylate(2l):*

Light yellow solid, 20% yield; mp: 191-196^°^C^ 1^H NMR (300 MHz, DMSO-*d*_6_): δ=1.5-1.8 (m, 6H,(adamantyl)), 1.9-2.3 (m, 9H, (adamantyl)), 6.15 (d, *J=*15.9 Hz, 1H, H-2’), 6.74 (d, *J=*8.1 Hz, 1H, ArCH=C*H*CO), 6.96-6.99 (dd, *J*_1_=8.1 Hz, *J*_2_=1.8 Hz, 1H, H-6’), 7.02 (d, *J*= 1.8 Hz, H-2’), 7.34 (d, *J*= 15.9 Hz, ArC*H*=CHCO), 9.16-9.58 (brs, 2H, -OH); ^13^C NMR (75 MHz, DMSO-*d*_6_): δ= 24.93, 25.79, 30.65, 33.77, 36.14, 41.46, 79.78, 115.14, 116.14, 116.36, 121.58, 126.11, 144.6, 145.79, 148.39, 166.05; IR (KBr): 3457, 3329, 2923, 2851, 1670cm^−1^; MS (*m/z*): 313 (M^+^); Anal. Calcd for (C_19_H_22_O_4_) calc. (%): C (72.59), H (7.05); found (%): C (70.84), H (6.78);

*(3r,5r,7r)-adamantan-1-yl)methyl 3,4-dihydroxybenzoate*

*(2m):*

White solid, 30% yield; mp: 212-217^°^C; ^1^H NMR (300 MHz, DMSO-*d*_6_): δ=1.59 (d,* J=*1.5 Hz, 6H, (adamantyl)), 1.63-1.74 (q, *J=*12 Hz, 6H, (adamantyl)), 1.91-2.5 (m, 3H, (adamantyl)), 4.04 (d, *J=*7.2 Hz, 1H, -CH_2_), 6.83 (d,* J=*11.2 Hz, 1H, H-5’), 7.30-7.37 (dd, *J*_1_*=*11.2 Hz, *J*_2_*=*2.8 Hz, H-6’), 7.39 (d, *J=*2.8 Hz, 1H, H-2’), 9.42-9.77 (brs, 2H, -OH);^ 13^C NMR (75 MHz, DMSO-*d*_6_): δ= 27.92, 33.56, 36.93, 39.26, 73.58, 115.82, 116.67, 121.22, 122.18, 145.53, 150.87, 166.14; IR (KBr): 3481, 3360, 2901, 2848, 1693 cm^−1^; MS (*m/z*): 302 (M^+^); Anal. Calcd for (C_18_H_22_O_4_) calc. (%): C (71.50), H (7.33); found (%): C (71.68), H (7.14);

*((3r,5r,7r)-adamantan-1-yl)methyl(E)-3-(3,4-dihydroxyphenyl)acrylate(2n)*

White solid, 35% yield; mp: 161-164^°^C;^ 1^H NMR (300 MHz, DMSO-*d*_6_): δ=1.55 (d, *J=*1.2 Hz, 6H, (adamantyl)), 1.61-1.72 (q, *J=*12.3 Hz, 6H, (adamantyl)), 1.92-2.0 (m, 3H, (adamantyl)), 3.74 (s, 2H, -CH_2_), 6.3 (d, *J=*15.9 Hz, 1H, H-2’), 6.77 (d, *J=*8.1 Hz, 1H, ArCH=C*H*CO), 7.01-7.05 (dd, *J*_1_= 8.1, *J*_2_=1.8, 1H, H-6’), 7.07 (d, *J*= 1.8, 1H, H-2’), 7.48 (d,* J*=15.6, 1H, ArC*H*=CHCO), 9.15-9.58 (brs, 2H, OH); ^13^C NMR (75 MHz, DMSO-*d*_6_): δ= 27.92, 33.56, 36.93, 39.21, 73.33, 114.53, 115.25, 116.16, 121.84, 126.03, 145.45, 145.48, 148.64, 167.12; IR (KBr): 3487, 3211, 2902, 2846, 1671 cm^−1^; MS (*m/z*): 328 (M^+^); Anal. Calcd for (C_20_H_24_O_4_) calc. (%): C (73.15), H (7.37); found (%): C (73.48), H (7.35);

*(2R)-1,7,7-trimethylbicyclo[2.2.1]heptan-2-yl 3,4-dihydroxybenzoate (2o):*

Colorless solid, 30% yield; mp: 40- 42^°^C. MS (*m/z*): 290 (M^+^) (18).

***General procedure for protection of hydroxyl group of phenolic acids***

*3,4-diacetoxybenzoic acid (2a* ^’^ *)*

To a solution of 3,4-dihydroxy benzoic acid (1 mmol, 0.236 g) and NaOH (14 mmol, 0.506 g) in H_2_O (1.5 ml), acetic anhydride (2 mmol, 0.154 g) was added dropwise at 10 ^°^C. The pH value of solution was mediated to 4-5 using diluted HCl. The white precipitate was filtered and washed with water. Compound **2a**^’^ was obtained as white solid with m.p: 160-162^°^C (lit. 158-160^°^C) (20).

*(E)-3-(4-acetoxy-3-methoxyphenyl) acrylic acid (2b* ^’^ *)*

(E)-3-(4-acetoxy-3-methoxyphenyl) acrylic acid **2b’** was prepared from (E)-3-(4-hydroxy-3-methoxyphenyl) acrylic acid (1 mmol, 0.154 g), NaOH (7 mmol, 0.253 g) in H_2_O (1.5 ml) and acetic anhydride (1 mmol, 0.102 g) following the same protocol as for 3,4-diacetoxybenzoic acid** 2a**^’^. Compound **2b**^’^ was obtained as white solid with m.p: 194-196 ^°^C (lit. 196-199^°^C) (21).

***General procedure for preparation of carboxylic amides (3a*** ^’^ ***-b*** ^’^ ***)***

To a solution of compound **2a**^’^ and **2b**^’^ (25 mmol) and triethylamine (25 mmol, 0.28 ml) in chloroform (15 ml), ethylchloroformate (25 mmol, 0.4 ml) was added dropwise at 0 ^°^C. The solution was stirred for 30 min at 0^°^C. This solution was added to a mixture of amantadine (27 mmol, 3.42 g) and triethyl amine (25 mmol, 0.28 ml) in chloroform (15 ml). The reaction mixture was stirred at room temperature for 30 min. Then, the mixture filtered and the solvent evaporated *in vaccum*. The residue was extracted with EtOAc (3×30 ml), washed with saturated aqueous NaHCO_3_ solution (30 ml), 0.1 M HCl (30 ml) and water (15 ml), dried over MgSO_4 _and the solvent evaporated. The residue was dissolved in mixture of K_2_CO_3 _(25 mmol, 3.45 g) in methanol (45 ml). The reaction mixture was refluxed for 4 hr. The mixture was neutralized by dilute aqueous HOAc solution and extracted by EtOAc (3×45 ml). The solvent was evaporated *in vaccum* and the crude product was purified by column chromatography on silica gel (eluent: chloroform/ methanol) (22).

*N-((3s,5s,7s)-adamantan-1-yl)-3,4-dihydroxybenzamide*

*(3a* ^’^ *)*

White solid, 45% yield; mp: 209-211^°^C;^ 1^H NMR (300 MHz, DMSO-*d*_6_): δ=1.650 (s, 6H, (adamantyl)), 2.04 (s, 9H, (adamantyl)), 6.72 (d,* J=*8.1 Hz, 1H, H-5’), 7.12 (d, *J=*8.1 Hz, 1H, H-6’), 7.2-7.5 (s, 2H, H-2’, -NH), 8.24 (brs, 2H, -OH);^ 13^C NMR (75 MHz, DMSO-*d*_6_): δ= 29.38, 36.61, 41.46, 51.59, 60.71, 72.74, 115.04, 115.63, 119.5, 127.65, 145.06, 148.37, 166.33; IR (KBr): 3521, 3506, 3376, 3255, 2908, 2848, 1630 cm^−1^; MS (*m/z*): 287 (M^+^); (M; Anal. Calcd for (C_17_H_21_NO_3_) calc. (%): C (71.06), H (7.37), N (4.87); found (%): C (70.68), H (7.08), N (4.20); 

*(E)-N-((3s,5s,7s)-adamantan-1-yl)-3-(4-hydroxy-3-methoxyphenyl)acrylamide (3b* ^’^ *)*

White solid, 43% yield; mp: 130-132^°^C; MS (*m/z*): 327 (M^+^) (23).

## Results

All esters were synthesized through estrification of monoterpenes with phenolic acids in the presence of N,N’-Dicyclohexylcarbodiimide (DCC) in a THF solvent (Figure 3). This method showed a better yield than esterification of acid chloride with alcohol. All phenolic acids and monoterpenes were purchased from commercial sources.

The inhibitory activity of all synthetic compounds against SLO (soybean 15-lipoxygenase: L1; EC 1,13,11,12) was determined using the peroxide formation method (13, 14). In this method, the basis of evaluation of lipoxygenase activity is measuring the concentration of fatty acid peroxide. All measurements were compared with 4-​methyl-​2-​(4-​methylpiperazinyl)pyrimido[4,​5-​b]benzothiazine (4-MMPB), one of the known lipoxygenase inhibitors (Table 1).

The radical scavenging ability of all synthetic compounds using stable free radicals (DPPH: 2,2-diphenyl-

1-picrylhydrazyl) has been previously measured (Table 1) (15). In this assessment, the DPPH bleaching potency of the synthetic compounds has been reported as IC_50_ values.

**Figure 2:**
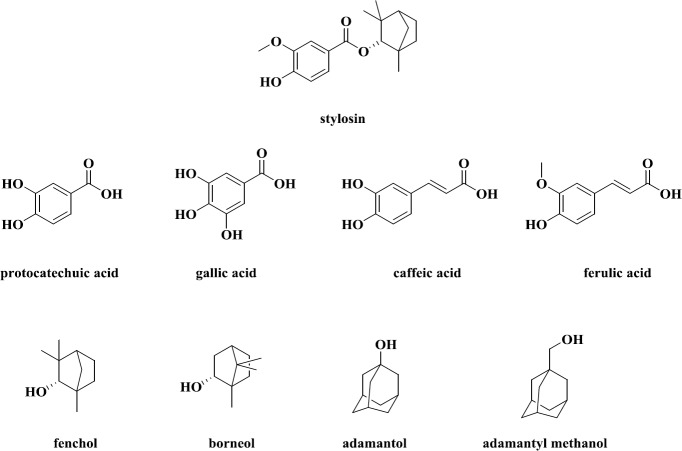
chemical structure of phenolic acids and monotrrpenoid alcohols

**Figure 3:**
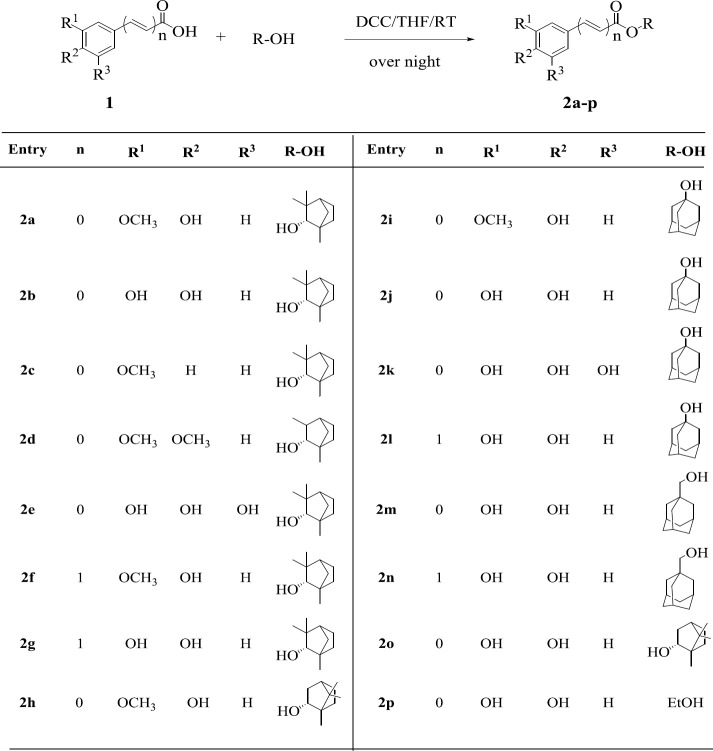
General procedure for the synthesis of compounds 2a-p

**Figure 4:**
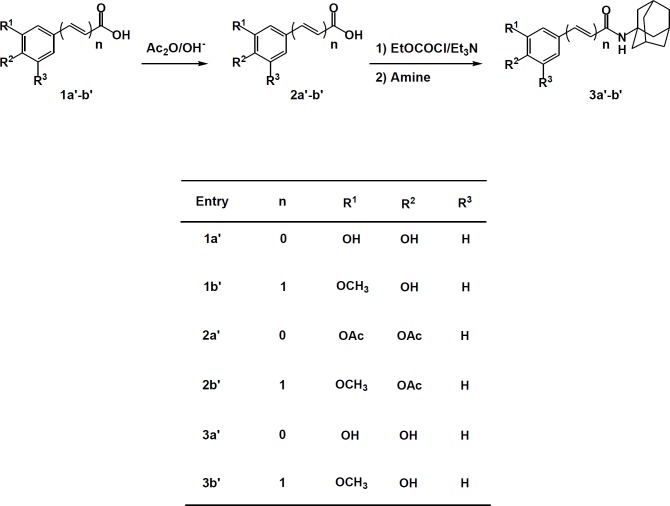
General procedure for the synthesis of compounds 2a-p

**Table 1:** IC_50_ values (µM) of both lipoxygenase inhibitory and 2,2-diphenyl-1-picrylhydrazyl (DPPH) bleaching activities of the synthetic compounds. Data are shown as ± SEM

**compound**	**DPPH bleaching**	**Lipoxygenase inhibition**	**compound**	**DPPH bleaching**	**Lipoxygenase inhibition**
2k	4.50 ± 0.19	2.28 ± 0.10	2a	> 500	40.1 ± 2.20
2l	18.0 ± 1.90	3.90 ± 0.61	2b	10.0±1.00	1.68 ± 0.61
2m	10.8 ± 0.67	3.57 ± 0.19	2c	> 500	> 500
2n	5.98 ± 0.53	2.70 ± 0.13	2d	> 500	> 500
2o	9.40 ± 0.81	0.78 ± 0.11	2e	3.46 ± 0.33	1.71 ± 0.41
2p	9.20 ± 0.45	6.50 ± 0.53	2f	> 500	194.0 ± 11.3
3a'	6.50 ± 0.43	5.40 ± 0.55	2g	10.9 ± 1.10	1.67 ± 0.22
3b'	5.05 ± 4.08	188.5 ± 14.0	2h	> 500	273.3 ± 11.3
4-MMPB	17.1 ± 2.00	38.5 ± 1.97	2i	> 500	164.0 ± 13.2
			2j	9.10 ± 0.79	0.95 ± 0.09

## Discussion

The stylosin **2a** showed well inhibitory activity against SLO (IC_50_ =40.1±2.2 μM). It is an ester of vanillic acid and fenchol (a monoterpene alcohol). A series of changes were made on both terpene and acidic moieties in order to synthesize appropriate inhibitors and study the structural effects on inhibition potency.

First, compound **2d** was synthesized by methylation of the hydroxyl group at the para-benzene ring of **2a**. The inhibition potency of **2d** was very low (IC_50_ > 500 μM). Also, **2c** with only one methoxy group at para position showed no inhibitory effect (IC_50_ > 500 μM).

Compound **2b** with two hydroxyl groups at both para and meta- positions (catechol) was synthesized. It showed a much better inhibitory effect than stylosin **2a** (IC_50 _= 1.68 ± 0.61 μM). Compound **2e** with three hydroxyl groups (galate) also showed much better inhibitory activity similar to **2b** (IC_50_ = 1.71 ± 0.41 μM). It was interesting that ethyl catechuate **2p** showed about 5-fold less inhibitory potency (IC_50_ = 6.50 ± 0.43 μM) versus **2b**, while the radical scavenging activity of both compounds was similar. It shows the role of the alcoholic moiety in lipoxygenase inhibition.

Other changes were performed in the acidic part of stylosin by replacing vanillic acid with frulic acid and caffeic acid. “Fenchyl frulate” (**2f**), which was synthesized by estrification of fenchol and fluric acid, showed a much weaker inhibitory effect versus stylosin (IC_50_ = 194.3 ± 11.3 μM). While Fenchyl Caffeate (**2g**) showed much better inhibitory potency (similar to **2b**) (IC_50_ = 1.67 ± 0.22 μM).

According to the recent studies on the inhibitory potency of the phenolic acids, it has been found that esters with hydrophobic rings such as borneol have a potent inhibitory effect on the lipoxygenase enzyme (16). Based on this, it was decided to change the monoterpene moiety of the stylosin to synthesize more potent inhibitors.

So, the monoterpene part of stylosin (fenchol) was replaced with borneol and adamantol. Compounds** 2h** and **2i** with borneyl and adamantly moieties were synthesized. They showed much lower inhibitory potency (compare to stylosin) with IC_50_ values of 273.3 ± 11.3 μM and 164.0 ± 13.2 μM, respectively.

Compound **2J** that was synthesized via the esterification of adamantol with protocatechuic acid, showed a better inhibitory activity compared to compound **2b** (IC_50 _= 0.95 ± 0.09 μM). Compound **2k** was synthesized by replacing fenchol with adamantol in the **2e** compound. Compared to the **2e**, **2k** had almost identical inhibition (IC_50_ = 2.28 ± 0.10 μM). 

Compound **2o** that was obtained by replacing the fenchol with borneol in compound **2b**, showed a better inhibitory activity compared to compound **2b** (IC_50 _= 0.78 ± 0.11 μM). Compound **2l** that was synthesized through the replacement of fenchol with adamantol in the compound **2g** showed a relatively lower inhibitory potency compared to compound **2g** (IC_50_ = 3.9 ± 0.61 μM).

Compound** 2m** was synthesized by the replacement of fenchol with ‘’adamantyl methanol’’ in compound **2b**, which showed a relatively lower inhibitory potency compared to compound **2b** (IC_50 _= 3.57 ± 0.19 μM), and compound **2n**, which was synthesized from esterification of adamantly methanol with caffeic acid, had a relatively lower inhibitory effect than the compound **2g** (IC_50 _= 2.70 ± 0.13 μM).

Although compound **2m** showed less potency for lipoxygenase inhibition in comparison with that of compound **2b**, its radical scavenging potency was not different from compound **2b**. By investigating the ability of radical scavenging of the synthetic compounds, it was found that compounds containing several hydroxyl groups on the benzene ring were more capable of free radical trapping. Also, by comparing compounds **2b** and **2g** wherein the compound **2g** has an added dual bond to compound **2b**, it was found that their free radical scavenging ability is the same as their lipoxygenase inhibitory activity.

Through investigations on the inhibitory effect of amide compounds against the soybean 15-lipoxygenase enzyme, it was proposed that amide compounds may have a better inhibitory effect than their constituents (17). So, it was decided to prepare amide compounds **3a’** and **3b’** and to further investigate their lipoxygenase inhibitory potency.

Amide compounds **3a’** and **3b’** were synthesized during the two reaction steps: reaction of amantadine with the phenolic acids protected by acetyl and then the hydrolysis of the acetyl group (Figure 4).

The lipoxygenase inhibitory potency of compound **3a’** was much better than stylosin (IC_50_ = 5.40 ± 0.55 μM). However, this amide compound showed a lower inhibitory activity compared to compound **2j**. Interestingly, the synthetic amides showed better radical scavenging activity than their steric homolog, which is probably due to weaker electronegativity effect of amide compared to ester portions. Compound **3b’** showed similar inhibitory potency compared to its homolog ester **2f** (IC_50_ = 188.5 ± 0.14 μM).

## Conclusion

By changing the alcohol and acyl portions of stylosin, it was found that electric properties play main role in lipoxygenase inhibition potency in contrast with steric features. Insertion of more reductive phenolic moiety such as catechuate and gallate can lead to more lipoxygenase inhibition activity.
